# Synergistic effects of sacituzumab govitecan and radiotherapy in preclinical models of colorectal cancer

**DOI:** 10.1093/gastro/goag101

**Published:** 2026-10-07

**Authors:** Ruowei Wang, Yingting Situ, Weifeng Wang, Yuanbin Liao, Jiahua He, Yuxiang Deng, Yanbo Xu, Songran Liu, Weili Zhang, Chi Zhou, Junzhong Lin, Weihao Li, Zhenhai Lu, Jianhong Peng

**Affiliations:** Department of Colorectal Surgery, State Key Laboratory of Oncology in South China, Collaborative Innovation Center for Cancer Medicine, Sun Yat-Sen University Cancer Center, 651 Dongfeng Road East, Guangzhou, Guangdong 510060, P. R. China; Department of Colorectal Surgery, State Key Laboratory of Oncology in South China, Collaborative Innovation Center for Cancer Medicine, Sun Yat-Sen University Cancer Center, 651 Dongfeng Road East, Guangzhou, Guangdong 510060, P. R. China; Department of Colorectal Surgery, State Key Laboratory of Oncology in South China, Collaborative Innovation Center for Cancer Medicine, Sun Yat-Sen University Cancer Center, 651 Dongfeng Road East, Guangzhou, Guangdong 510060, P. R. China; Department of Colorectal Surgery, State Key Laboratory of Oncology in South China, Collaborative Innovation Center for Cancer Medicine, Sun Yat-Sen University Cancer Center, 651 Dongfeng Road East, Guangzhou, Guangdong 510060, P. R. China; Department of Pathology, State Key Laboratory of Oncology in South China, Collaborative Innovation Center for Cancer Medicine, Sun Yat-Sen University Cancer Center, 651 Dongfeng Road East, Guangzhou, Guangdong 510060, P. R. China; Department of Thyroid and Breast Surgery, Shenzhen Second People’s Hospital, The First Affiliated Hospital of Shenzhen University, Shenzhen 518035, P. R. China; Department of Thyroid Surgery, Zhujiang Hospital of Southern Medical University, Guangzhou, Guangdong 510282, P. R. China; Department of Pathology, State Key Laboratory of Oncology in South China, Collaborative Innovation Center for Cancer Medicine, Sun Yat-Sen University Cancer Center, 651 Dongfeng Road East, Guangzhou, Guangdong 510060, P. R. China; Department of Colorectal Surgery, State Key Laboratory of Oncology in South China, Collaborative Innovation Center for Cancer Medicine, Sun Yat-Sen University Cancer Center, 651 Dongfeng Road East, Guangzhou, Guangdong 510060, P. R. China; Department of Colorectal Surgery, State Key Laboratory of Oncology in South China, Collaborative Innovation Center for Cancer Medicine, Sun Yat-Sen University Cancer Center, 651 Dongfeng Road East, Guangzhou, Guangdong 510060, P. R. China; Department of Colorectal Surgery, State Key Laboratory of Oncology in South China, Collaborative Innovation Center for Cancer Medicine, Sun Yat-Sen University Cancer Center, 651 Dongfeng Road East, Guangzhou, Guangdong 510060, P. R. China; Department of Colorectal Surgery, State Key Laboratory of Oncology in South China, Collaborative Innovation Center for Cancer Medicine, Sun Yat-Sen University Cancer Center, 651 Dongfeng Road East, Guangzhou, Guangdong 510060, P. R. China; Department of Colorectal Surgery, State Key Laboratory of Oncology in South China, Collaborative Innovation Center for Cancer Medicine, Sun Yat-Sen University Cancer Center, 651 Dongfeng Road East, Guangzhou, Guangdong 510060, P. R. China; Department of Colorectal Surgery, State Key Laboratory of Oncology in South China, Collaborative Innovation Center for Cancer Medicine, Sun Yat-Sen University Cancer Center, 651 Dongfeng Road East, Guangzhou, Guangdong 510060, P. R. China

**Keywords:** colorectal cancer, sacituzumab govitecan, radiotherapy, synergistic antitumor effect, preclinical model

## Abstract

**Background:**

Although radiotherapy (RT) remains the standard of care for locally advanced colorectal cancer (CRC), intrinsic and acquired resistance frequently compromise long-term outcomes. Sacituzumab govitecan (SG)—a TROP2-directed antibody–drug conjugate—offers a targeted platform for intracellular SN-38 delivery. The aim of the present preclinical study was to investigate whether SG enhances radiosensitivity in CRC, thereby providing a preclinical rationale for the clinical evaluation of SG-based combination strategies.

**Methods:**

We evaluated SG–RT synergy across CRC cell lines with differential TROP2 expression (SW480, DLD-1 vs SW620, HCT116). In mechanistic studies, clonogenic assays, flow cytometry, cell counting kit-8, Western blot, and comet assays were utilized to assess cell survival, apoptosis, and DNA damage. TROP2 was knocked down or overexpressed to validate its role in radiosensitization. *In vivo* efficacy was evaluated by using cell-line-derived xenograft (CDX) and patient-derived xenograft (PDX) mouse models. Immunohistochemistry for cleaved caspase-3 (CASP3), phosphorylated histone H2AX (γ-H2AX), and Ki-67 were used to assess treatment-induced biological effects.

**Results:**

In TROP2-high CRC cell lines (SW480, DLD-1), combined SG and RT markedly inhibited proliferation, increased apoptosis, and amplified DNA double-strand breaks, as evidenced by elevated γ-H2AX expression and comet assay tail moments. Knockdown of TROP2 abrogated this synergy, whereas TROP2 overexpression restored it. In CDX and PDX models, SG combined with RT significantly suppressed tumor growth, increased cleaved CASP3 and γ-H2AX expression, and reduced Ki-67 without exacerbating systemic weight loss.

**Conclusion:**

Our findings identify SG as a potent radiosensitizer, providing a strong rationale for integrating SG into neoadjuvant RT regimens for locally advanced CRC.

## Introduction

Colorectal cancer (CRC) poses a formidable global health challenge, with rectal carcinoma accounting for over half of all cases in China, a subset often linked to poorer outcomes than those with colon cancer [1, 2]. Although neoadjuvant chemoradiotherapy (nCRT) is the standard treatment for locally advanced rectal cancer (LARC), patient responses are remarkably heterogeneous [3–6]. Our previous retrospective data indicated that >50% of patients failed to achieve significant tumor regression [7]. As the degree of tumor regression correlates with overall survival, enhancing radiosensitivity remains an unmet need in LARC management.

The topoisomerase I inhibitor irinotecan is a promising radiosensitizer. The Phase III CinClare trial led by Zhang *et al*. [8] demonstrated that adding irinotecan to capecitabine-based chemoradiotherapy (CapIriRT) doubled the pathological complete response rate (from 15% to 30%, *P *= 0.001). However, its clinical utility is hampered by excessive Grade 3–4 toxicities (38% vs 6%, *P *< 0.001). Sacituzumab govitecan (SG) (IMMU-132) is a next-generation antibody–drug conjugate (ADC) linking SN-38, the active metabolite of irinotecan, to a humanized anti-TROP2 antibody [9]. By enabling targeted delivery, SG maintains the DNA-damaging potency of SN-38, suggesting improved tolerability and feasibility for combination therapy.

Radiotherapy (RT) induces DNA double-strand breaks [10], whereas SN-38 inhibits topoisomerase I-mediated DNA repair [11]. The combination of these effects may amplify DNA damage and apoptosis, thereby achieving synergistic tumor control. Moreover, radiation enhances tumor antigen exposure and drug accessibility [12], potentially promoting the TROP2-mediated uptake of SG into CRC cells. This mechanism could increase intratumoral SN-38 accumulation and augment its therapeutic efficacy while minimizing off-target toxicity. Despite these theoretical advantages, no studies have investigated the radiosensitizing effect of SG in CRC.

In this study, we evaluated whether SG synergistically enhances radiosensitivity in a TROP2-dependent manner, providing a preclinical basis for novel combined-modality strategies for CRC.

## Materials and methods

### Cell cultures and treatment

CRC cells (SW480, SW620, DLD-1, and HCT116) were purchased from the American Type Culture Collection and cultured in Roswell Park Memorial Institute (RPMI) 1640 (SW620 and DLD-1) or Dulbecco's Modified Eagle Medium (DMEM) (SW480 and HCT116) supplemented with 10% fetal bovine serum (10099141; Gibco), 100 IU/mL of penicillin, and 100 μg/mL of streptomycin at 37°C in 5% CO_2_. All cell lines used in our study were authenticated via short tandem repeat (STR) profiling and tested negative for mycoplasma contamination before the experiments. Cells were irradiated by using an RS-2000-PRO-225 Biological Irradiator.

### Cell counting kit-8 assay and synergy analysis

For the proliferation assay, CRC cells were seeded in 96-well plates at a density of 4,000 cells per well. The following day, the cells were treated with SG alone, radiation alone, or a combination of SG and radiation at varying doses. After 2 days, cell counting kit-8 (DOJINDO) was added to the wells and incubated at 37°C for 2 h. Absorbance was measured at 450 nm and growth curves were generated to determine the growth rates.

Drug–radiation interaction effects were evaluated using the Zero Interaction Potency (ZIP) model implemented in R package “synergyfinder” (version 3.6.3). ZIP synergy scores were calculated based on normalized viability data derived from dose–response matrices. According to standard criteria, ZIP scores of >10 indicate synergistic interactions, scores of between −10 and 10 indicate additive effects, and scores of less than −10 indicate antagonistic interactions.

### Apoptosis assay

For the cell apoptosis assay, CRC cells were treated with SG alone, radiation alone, or SG plus radiation at varying doses for 2 days. Subsequently, the cells were harvested and stained with ANXA5/annexin V-FITC and propidium iodide (PI) by using an Annexin V-FITC/PI staining kit (556547; BD Pharmingen™). Following a 15-min incubation at room temperature, the cells underwent analysis via flow cytometry.

### Colony formation assay

Cells were seeded in six-well plates (2,000 cells per well) with three replicates and treated with SG alone, radiation alone, or SG plus radiation at varying doses on the following day for 2 days. The cells were cultured for another 10 days with complete medium at 37°C with 5% CO_2_. Cells were then fixed with methanol and stained with crystal violet, and the colonies of each well were counted.

### Western blot analysis

Cells were lysed by using NP-40 (P0013F; Beyotime) for 15 min, followed by centrifugation at 13,000 ×* g* for 15 min at 4°C. Equivalent quantities of total protein lysates were subjected to 10% sodium dodecyl sulfate–polyacrylamide gel electrophoresis (SDS–PAGE) and subsequently transferred to polyvinylidene difluoride (PVDF) membranes. The membranes were blocked with 5% BSA (BS114-500 g; Biosharp) in TBS (BL602A; Biosharp) containing 0.1% Tween-20 (ST825-100 mL; Beyotime) for 1 h and then incubated with the appropriate specific primary antibody overnight at 4°C. The primary antibodies utilized in this study include anti-TROP2 (ab214488; Abcam), anti-Phospho-H2A.X (GB111841-100; Servicebio), anti-Vinculin (ab129002; Abcam), anti- Beta Actin (81115–1-RR; Proteintech), and anti-ABCG2 (F0718; Selleck). The membranes were washed and incubated for 1 h at room temperature with horseradish peroxidase (HRP)-linked secondary antibodies (SA00001-2; Proteintech).

### Comet assay

Comet assays were performed by using a commercial kit (KTA3040; Abbkine). Cells were harvested and combined with agarose, and then layered on three-well comet slides that had been pre-coated with normal agarose. The slides were then lysed for 30 min at 4°C in lysis buffer and incubated for 30 min in pre-chilled alkaline solution, followed by electrophoresis for 20 min at 30 V and 200 mA. Afterward, all slides were neutralized with 0.4 mM Tris-HCl solution at 4°C for 10 min. DNA staining was performed with 1 × PI dye for 10 min at room temperature and protected from light, followed by washing with phosphate buffered saline (PBS). Samples were imaged by using a microscope and analysed by using a comet assay software project.

### *In vivo* therapeutic study

CRC cell-line-based xenograft (CDX) and patient-derived xenograft (PDX) models were established for *in vivo* evaluation. All mice were housed under specific pathogen-free conditions. All procedures, following the National Institutes of Health Guide for the Care and Use of Laboratory Animals (NIH Publications No. 8023, revised 1978), were approved by the Sun Yat-sen University Animal Care and Use Committee (L102012025010G; Guangzhou, China). Six-week-old female BALB/c nude mice were purchased from the Vital River Laboratory (Beijing, China). Sample sizes for animal experiments were determined based on previous similar preclinical therapeutic studies, preliminary pilot observations, and ethical considerations, to minimize animal use while maintaining sufficient statistical sensitivity to detect biologically meaningful differences between treatment groups, in accordance with ARRIVE guidelines [13].

SW480 cells (5 × 10^5^) were suspended in 100 μL of PBS and subcutaneously injected into the right flank of each mouse (five mice/group, randomly divided into four groups). One week later, the mice were intraperitoneally injected with the following treatments: SG alone (25 mg/kg), radiation alone (3 Gy), SG plus radiation, or mock treatment. Radiation treatments were delivered by using a small animal radiation research platform (RS-2000-PRO-225; Radsource). SG was suspended in PBS and intraperitoneally injected into mice in the indicated treatment group. For combination treatment, SG was administered 1 h after irradiation every 3 days for four cycles. This schedule was selected based on the rationale that RT-induced DNA damage may sensitize tumor cells to subsequent SN-38-mediated topoisomerase I inhibition, thereby maximizing the temporal overlap between radiation-induced injury and SG-mediated cytotoxic activity [14, 15]. In addition, a short interval between irradiation and SG administration was introduced to minimize potential interference between SG treatment and the sodium pentobarbital anesthesia used during the irradiation procedures for animal immobilization.

The PDX models were initially generated by using fresh tumor samples from a patient with CRC. TROP2 expression in the PDX tumors was evaluated by using Western blot analysis and confirmed to be highly expressed (Supplementary Fig. S1). This study received approval from the Ethics Committee of Sun Yat-sen University Cancer Center (approval number: B2024-652–02). Primary CRC samples were sectioned into fragments measuring 1 mm^3^ and subsequently implanted subcutaneously into the dorsal flank of the mice, constituting the first generation (F0). Upon reaching a suitable volume, the tumors were excised, segmented into equal portions, and re-implanted subcutaneously into the mice, forming the second generation (F1). Once the tumors became palpable, the tumor-bearing mice were randomly allocated into four groups as previously described, with five mice per group.

The tumor-growth curve was measured every 3 days for 4–5 weeks and the volume of the tumor was calculated as length × width^2^ × 1/2. At the end of the study, the tumors were surgically removed from the sacrificed mice. The tumor growth inhibition (TGI) for each treatment group was calculated as: $\text{TGI}=1-\frac{{\left(V_{t}-V_{0}\right)}_{\text{treatment}}}{{\left(V_{t}-V_{0}\right)}_{\text{control}}}$. The synergy ratio was calculated based on the Bliss independence model:


$$\text{Synergy}\;\text{ratio}\;=\;\frac{TGI_{SG+RT}}{TGI_{SG}+TGI_{RT}-TGI_{SG}\times TGI_{RT}}$$

## Immunohistochemistry

Immunohistochemistry (IHC) analysis was performed by using paraffin-embedded tumor specimens in an *in vivo* therapeutic study. Primary antibodies against anti-Phospho-H2A.X (GB111841-100; Servicebio), anti-Ki-67 (ab16667; Abcam), and cleaved caspase-3 (CASP3) (9662; Cell Signaling Technology) were used for IHC staining. IHC staining was evaluated by two independent gastrointestinal pathologists who were blinded to the treatment assignments. The final IHC score was calculated based on the percentage of positively stained cells.

### Statistical analysis

All data were presented as the mean ± SD from at least three independent experiments. Statistical analyses were performed by using Prism (version 10.0; GraphPad Software, USA). Statistical tests used in this study included the two-way analysis of variance (ANOVA) followed by Tukey’s multiple-comparison test. This approach was applied to reduce the risk of false-positive findings across multiple comparisons. For *in vitro* combination studies, synergistic interactions between SG and RT were evaluated by using the ZIP model implemented in R package “synergyfinder” (version 3.6.3). A *P* value of <0.05 was considered statistically significant. *P* values are shown in the figures and/or legends with asterisks.

## Results

### SG synergizes with RT in a TROP2-dependent manner in CRC cells

The radiosensitizing effect of SG was closely associated with TROP2 expression. ZIP synergy analysis demonstrated positive synergy scores (ZIP > 10) in TROP2-high CRC cells (SW480 and DLD-1; Fig. 1A and C, and Supplementary Fig. S2) that were lost upon TROP2 silencing (Fig. 1B and D). In contrast, only additive effects (ZIP scores of between −10 and 10) were observed in TROP2-low cell lines (SW620 and HCT116; Fig. 1A and E, and Supplementary Fig. S3), whereas enforced TROP2 overexpression in SW620 cells restored the SG-mediated radiosensitization (Fig. 1B and F). At the lowest synergistic doses (3 nM of SG for SW480, 20 nM for DLD-1, and 4 Gy of radiation), the combination significantly increased apoptosis, as measured by using flow cytometry (*P *< 0.001; Fig. 2A  and B), and suppressed clonogenic survival compared with either treatment alone (*P *< 0.05; Fig. 2C  and D). These findings suggest that TROP2 is essential for SG-induced radiosensitization in CRC cells.

**Figure 1:**
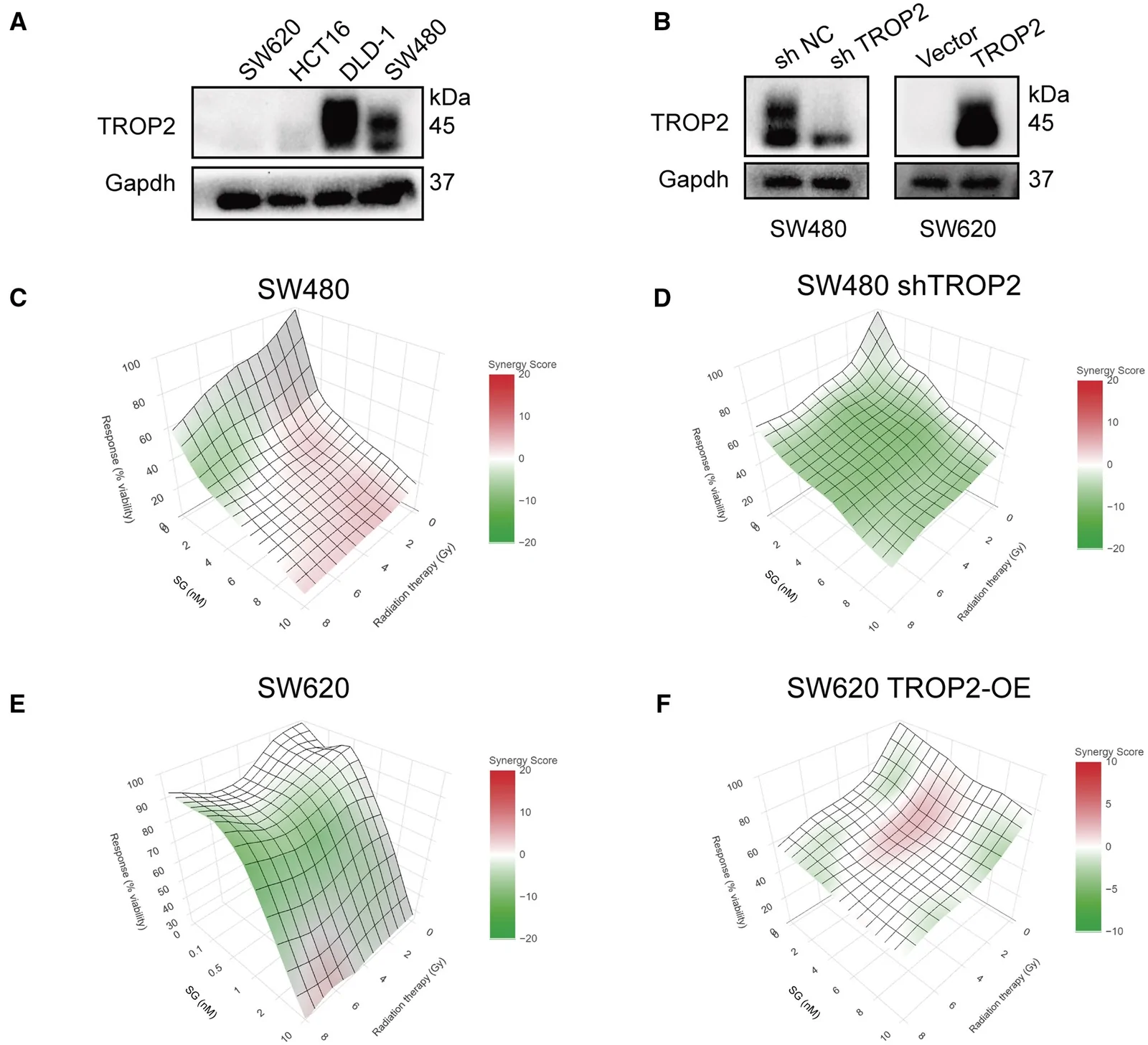
SG synergizes with RT in CRC cells through a TROP2-dependent mechanism. (A) Western blot analysis of TROP2 protein levels in CRC cell lines (SW620, HCT116, DLD-1, and SW480). (B) Validation of TROP2 knockdown in SW480 (shTROP2) and overexpression in SW620 (OE-TROP2) via Western blot. (C) ZIP synergy plots demonstrating additivity or synergy for the combination of SG with RT in SW480 cells. (D) ZIP synergy plots demonstrating additivity or synergy for combination of SG with RT in TROP2-knockdown (shTROP2) SW480. (E) ZIP synergy plots demonstrating additivity or synergy for the combination of SG with RT in SW620 cells. (F) ZIP synergy plots demonstrating additivity or synergy for the combination of SG with RT in TROP2-overexpressed (TROP2-OE) SW620 cells. NC, negative control.

**Figure 2:**
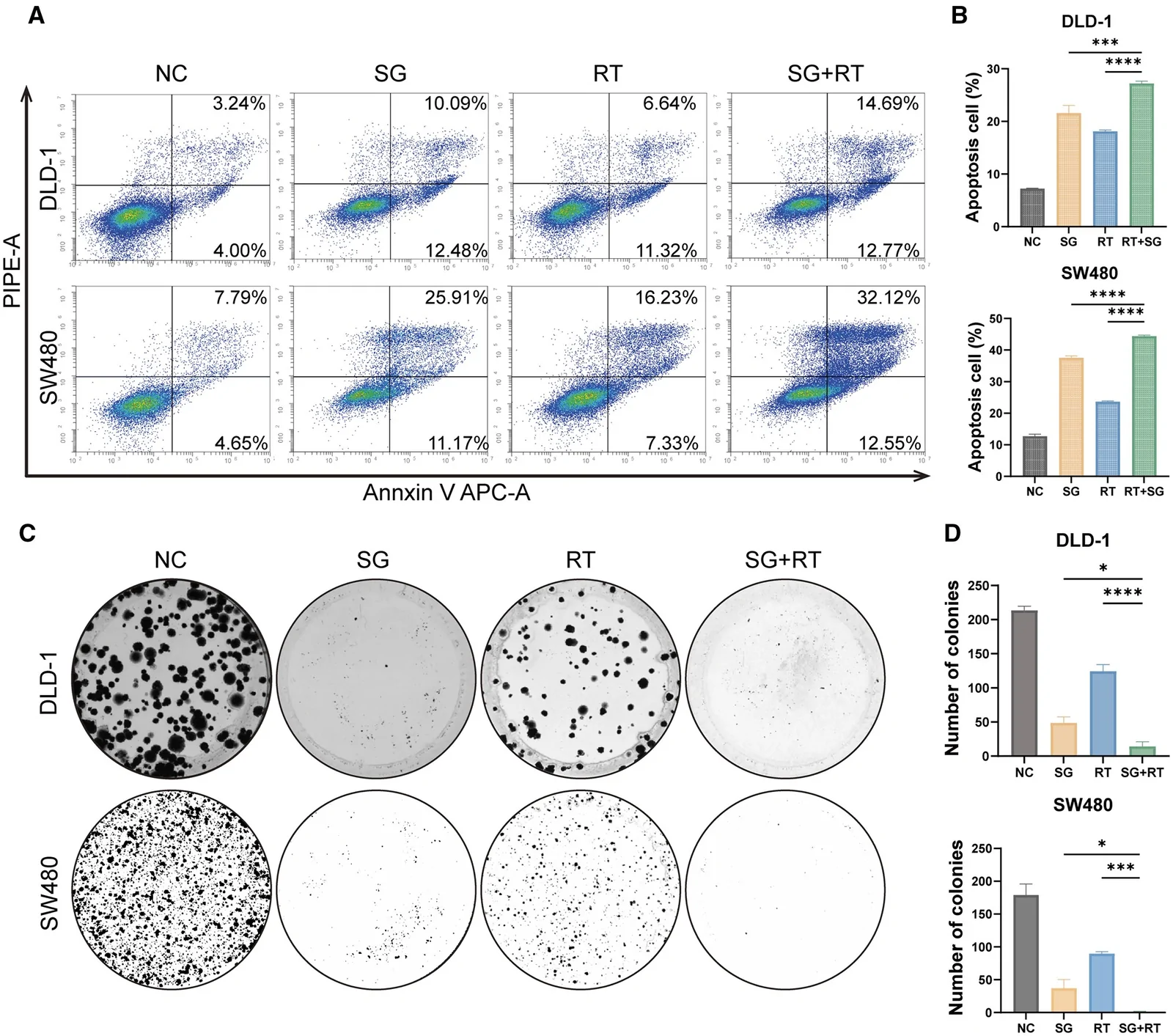
SG combined with RT in CRC cells in a TROP2-dependent manner. (A) Flow cytometric analysis of apoptosis in DLD-1 and SW480 cells treated with SG, RT, or both. (B) Quantification of the apoptotic rate of cells in (A). (C) Clonogenic survival assays showing decreased colony formation upon combination treatment. (D) Quantification of colony formation in (C). Data are representative of three independent experiments. All data represent mean ± SD. Two-way ANOVA was utilized. *P < 0.05, **P < 0.01, ***P < 0.001, ****P < 0.0001. NC, negative control.

### SG enhances radiation-induced DNA damage more effectively than SN-38

To determine whether the ADC structure provides an advantage over the free payload, we compared SG and SN-38 at equivalent concentrations. At escalating radiation doses, SG + RT suppressed cell proliferation more effectively than SN-38 + RT (*P *< 0.01; Fig. 3).

**Figure 3:**
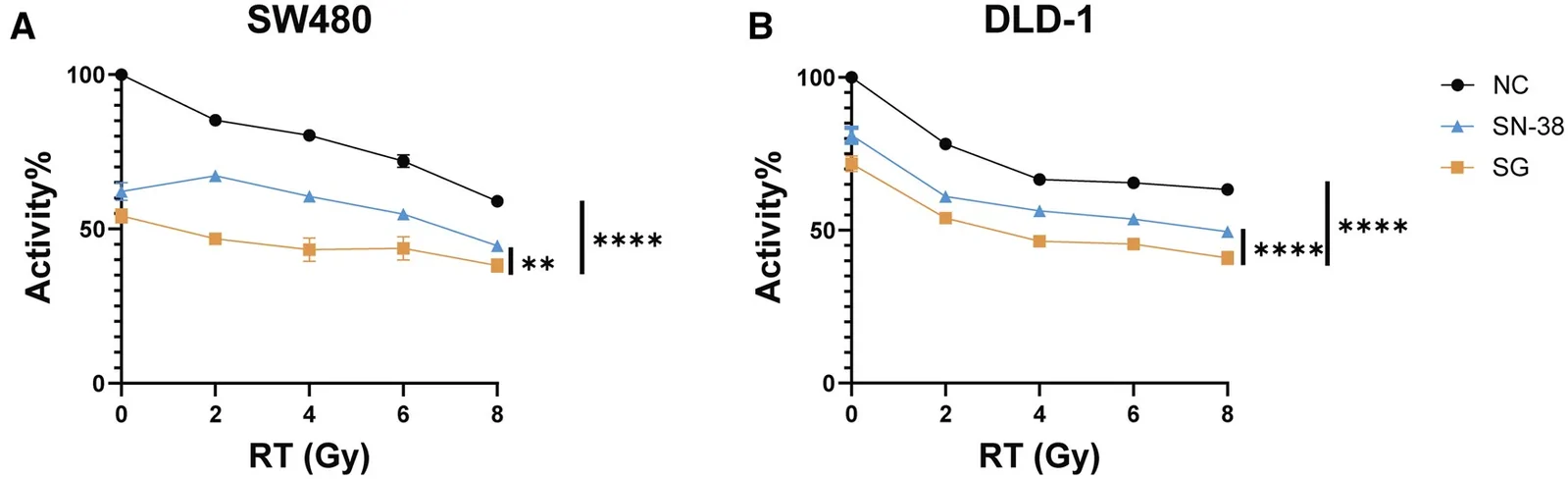
SG exhibited stronger growth inhibition than did SN-38 across all radiation doses. (A, B) Cell viability of (A) SW480 and (B) DLD-1 cells after treatment with SG or SN-38 combined with increasing radiation doses. SW480 cells were treated with 3 nM of SG or 3 × 7.6 nM of SN-38. DLD-1 cells were treated with 20 nM of SG or 20 × 7.6 nM of SN-38. Data are representative of three independent experiments. All data represent mean ± SD. Two-way ANOVA was utilized. *P < 0.05, **P < 0.01, ***P < 0.001, ****P < 0.0001. NC, negative control.

To measure the combined genotoxic effect, we analysed phosphorylated histone H2AX (γ-H2AX) induction and comet assays to detect DNA damage. SG + RT treatment enhanced γ-H2AX foci formation compared with SN-38 + RT or other treatments alone (*P *< 0.0001; Fig. 4A–C). This was also supported by comet assays (*P *< 0.05; Fig. 4D–F, which revealed more extensive DNA damage in the SG + RT-treated cells, confirming the superior genotoxic potency of SG over equimolar SN-38 in the context of radiation.

**Figure 4:**
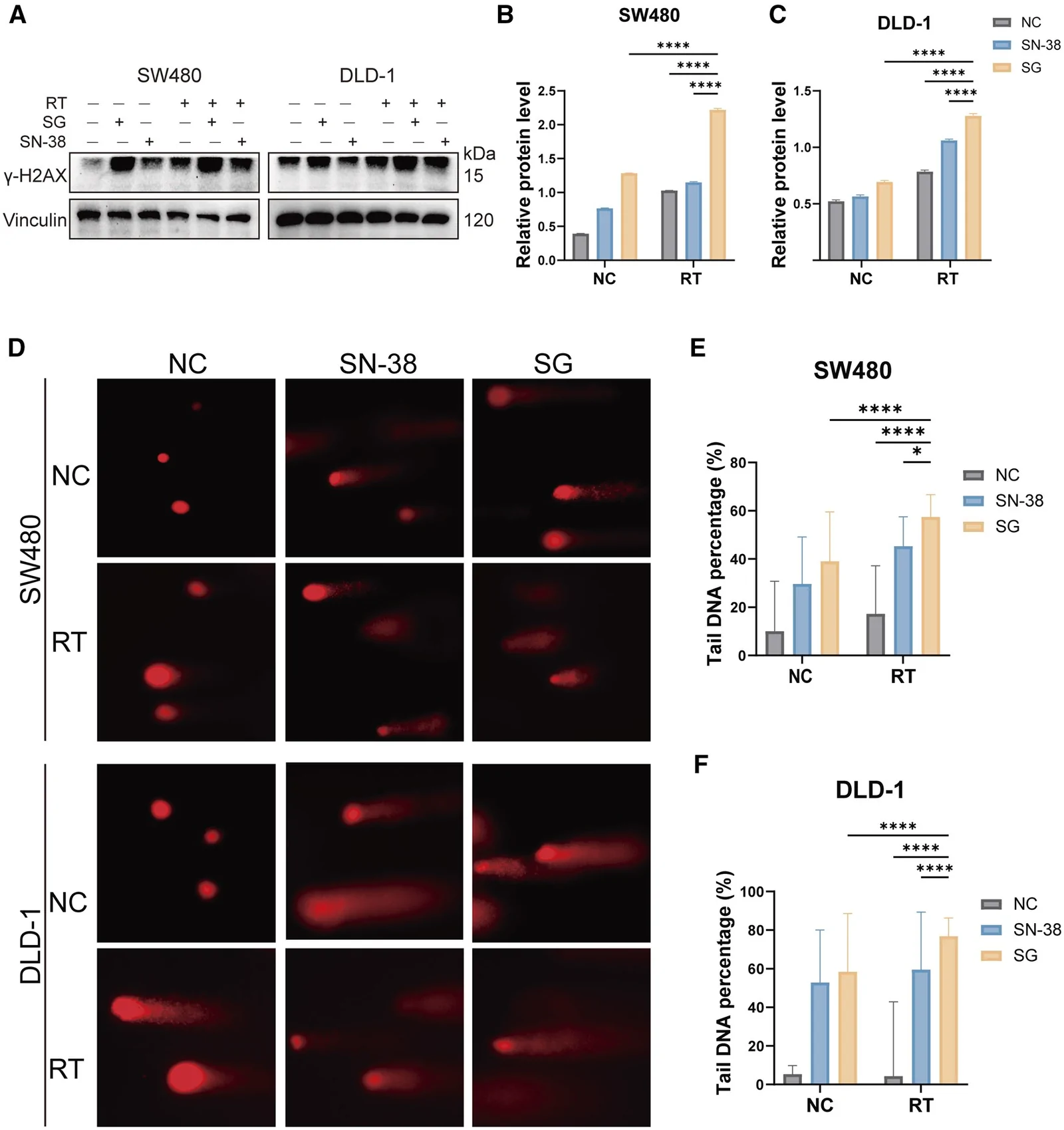
SG enhanced radiation-induced DNA damage more effectively than did SN-38. (A) Western blot detection of γ-H2AX levels after the indicated treatments. (B, C) Quantification of γ-H2AX expression in (A). (D) Comet assay illustrating DNA damage in SW480 and DLD-1 cells treated with SG or SN-38 in combination with RT. (E, F) Quantification of comet tail moment in (D). Data are representative of three independent experiments. All data represent mean ± SD. Two-way ANOVA was utilized. *P < 0.05, **P < 0.01, ***P < 0.001, ****P < 0.0001. NC, negative control.

### Combined SG and RT exert potent antitumor effects *in vivo*

We next examined the combined antitumor activity of SG and RT *in vivo* by using SW480-derived CDX and PDX models established in nude mice. The experimental timeline of the *in vivo* study is shown in Fig. 5A.

**Figure 5:**
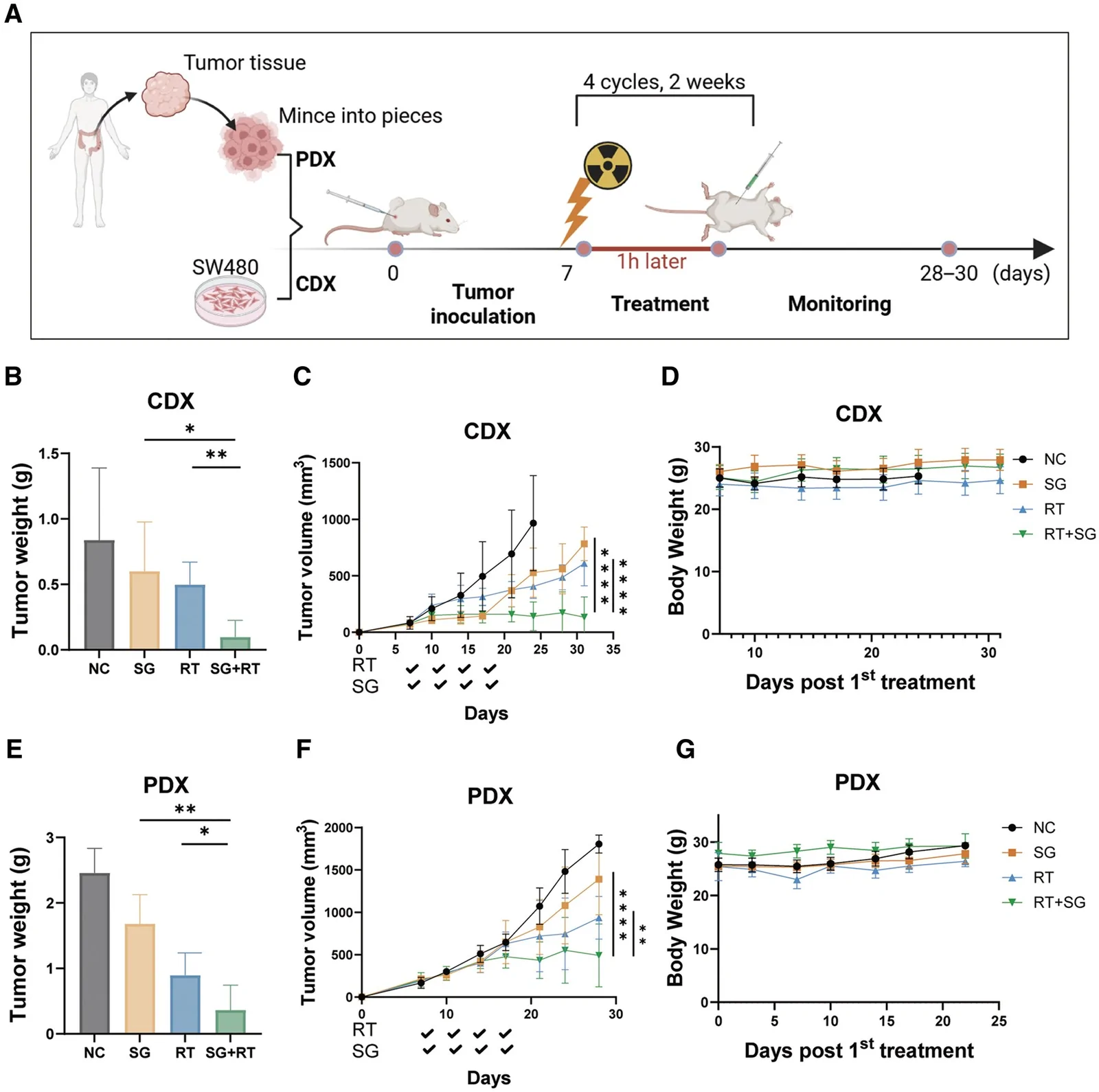
Combined treatment with SG and RT suppressed tumor growth in CRC xenograft models. (A) Experimental timeline of the *in vivo* study: when tumors reached 100 mm³, the mice were randomized into four groups: control (PBS), SG alone, RT alone, and combination therapy. Mice received 3 Gy of localized irradiation and 25 mg/kg of SG intraperitoneally; for the combination group, SG was administered 1 h post-irradiation every 3 days for four cycles. Created in BioRender. Wang, R. (2026). https://BioRender.com/usxvjcy. (B, E) Final tumor weights in the CDX and PDX models at the study endpoint. (C, F) Tumor-growth curves showing significantly reduced tumor volume in the combination group compared with that in the SG- or RT-alone groups. (D, G) Mouse body-weight changes during treatment. Mice were treated with 25 mg/kg of SG and 3 Gy of RT every 3 days for four cycles. All data represent mean ± SD. Two-way ANOVA was utilized. *P < 0.05, **P < 0.01, ***P < 0.001, ****P < 0.0001. NC, negative control.

Tumor growth was significantly delayed in the combination group compared with that in either monotherapy group (CDX: *P *< 0.0001; PDX: *P *< 0.01; Fig. 5B–G and Supplementary Figs S4 and S5). Significant differences between the RT and SG + RT groups were observed from Day 21 onward, with sustained tumor suppression at subsequent time points (adjusted *P *< 0.01 for multiple comparisons). Effect sizes and corresponding 95% confidence intervals demonstrated robust treatment-associated differences, particularly at later stages of treatment (Supplementary Tables S1 and S2). Notably, in CDX models, tumor volumes in the combination group remained <500 mm³ throughout the 31-day observation period, with a mean tumor volume of 133.3 mm^3^ at the endpoint. In contrast, tumors in the control and monotherapy groups exceeded 500 mm³ within 19.4 ± 3.9 days (PBS), 26.4 ± 3.9 days (SG), and 28.4 ± 2.9 days (RT). The combination yielded a TGI of 93.4% in CDX models and 77.1% in PDX models, whereas the TGIs for SG alone were 21.2% (CDX) and 24.4% (PDX), and for RT alone were 25.4% (CDX) and 51.0% (PDX). Based on the Bliss independence model, the synergy ratios were 2.26 (CDX) and 1.22 (PDX), indicating synergistic interactions between SG and RT. No significant body-weight loss or behavioral toxicity was observed during the treatment (Fig. 5H and I).

IHC staining revealed elevated levels of cleaved CASP3 and γ-H2AX and reduced Ki-67 expression in tumors from the combination group (Fig. 6), indicating enhanced apoptosis and DNA damage *in vivo*.

**Figure 6:**
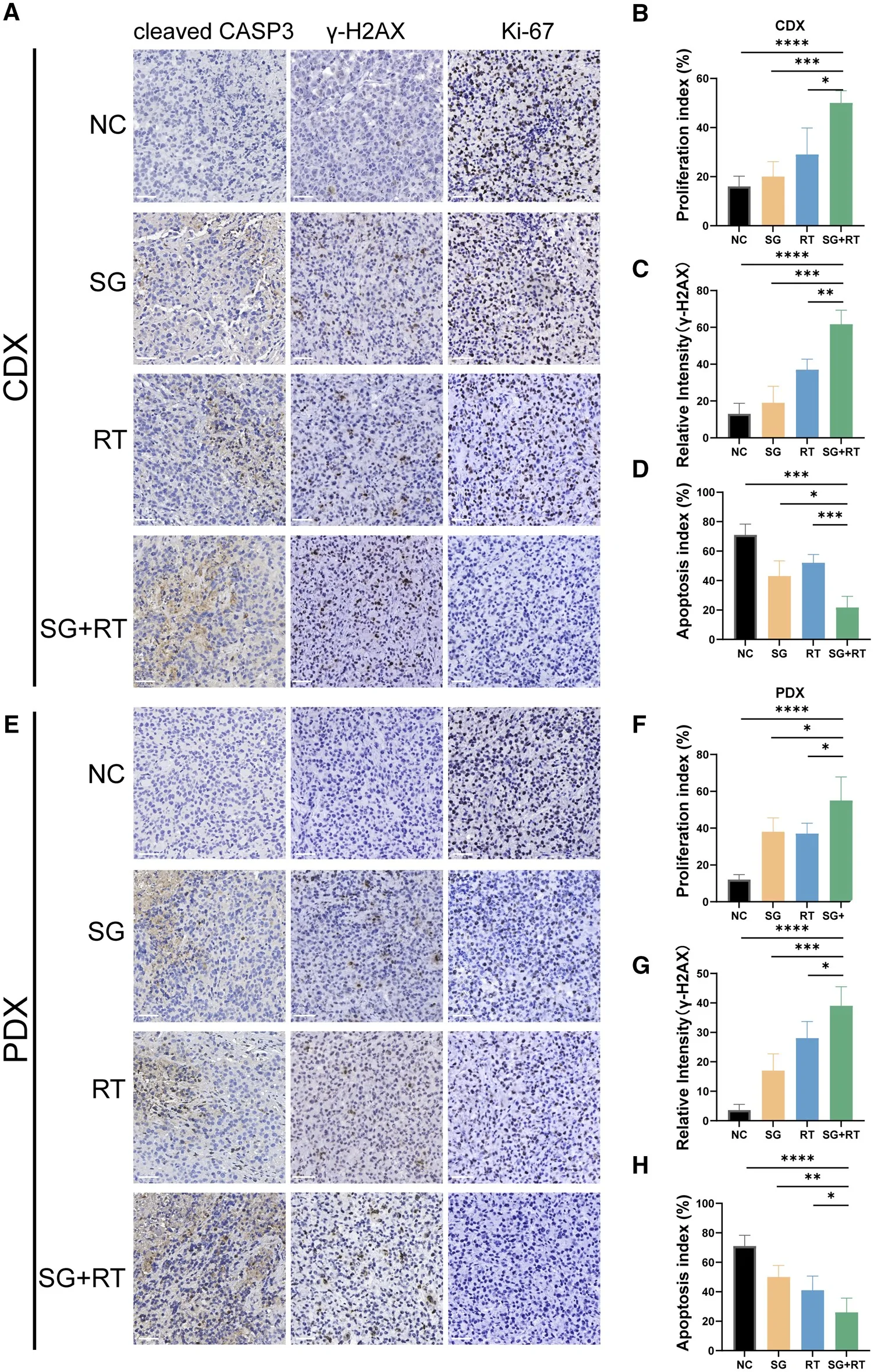
Immunohistochemical analysis of CRC xenografts following combined SG and RT. (A) Representative IHC staining of cleaved CASP3, γ-H2AX, and Ki-67 in CDX tumor tissues treated with PBS control, SG, RT, or combination therapy (SG plus RT) (scale bar: 50 μm). (B–D) Quantification of apoptosis ((B) cleaved CASP3), DNA damage ((C) γ-H2AX), and proliferation ((D) Ki-67) in the indicated groups of CDX models. Each index was calculated as the percentage of the marker-positive cells. (E) Representative IHC staining of cleaved CASP3, γ-H2AX, and Ki-67 in PDX tumor tissues treated with PBS control, SG, RT, or combination therapy (SG plus RT) (scale bar: 50 μm). (F–H) Quantification of apoptosis ((F) cleaved CASP3), DNA damage ((G) γ-H2AX), and proliferation ((H) Ki-67) in the indicated groups of PDX models. Each index was calculated as the percentage of marker-positive cells. All data represent mean ± SD. *P < 0.05, **P < 0.01, ***P < 0.001, ****P < 0.0001. NC, negative control.

## Discussion

These data establish SG as a TROP2-dependent radiosensitizer in CRC. The observation that TROP2 levels govern this synergy is pivotal, suggesting the utility of the receptor in patient stratification.

The mechanism appears to involve a “one–two punch”: RT induces DNA double-strand breaks, while SN-38 released from the SG further increases the DNA damage burden and impairs the resolution of radiation-induced DNA lesions through topoisomerase I inhibition [16]. Similar synergistic mechanisms have been observed in irinotecan–RT combinations in CRC [8, 17, 18], further supporting the translational rationale for this approach. Although enhanced DNA damage accumulation and apoptosis were consistently observed following the combination treatment, the precise molecular pathways underlying SG-mediated radiosensitization require further investigation.

Our comparative analysis revealed that SG exhibited stronger radiosensitizing effects than did equimolar SN-38. Although targeted delivery explains much of this effect, radiation may enhance ADC recruitment by upregulating TROP2 surface expression. Indeed, ionizing radiation is known to induce multiple cell-surface adhesion and immune-recognition molecules in epithelial cancers, suggesting a similar mechanism for TROP2 [12, 19, 20]. To preliminarily explore whether RT alters TROP2 expression, Western blot analysis was performed following treatment with SG, RT, or the combination. Modest increases in TROP2 expression were observed across multiple treatment conditions; however, no RT-specific or combination-specific upregulation was identified under the tested conditions (Supplementary Fig. S6). SG may also exert antitumor activity through ADC-specific mechanisms such as the SN-38-mediated bystander effect. As RT can modulate drug-transporter expression, we examined ABCG2, a key SN-38 efflux transporter, after irradiation (Supplementary Fig. S7). However, no significant changes in ABCG2 expression were observed across the tested radiation doses, suggesting that altered ABCG2-mediated SN-38 retention is unlikely to be a major contributor to the enhanced efficacy of SG plus RT in our model. The potential roles of other transporters, tumor microenvironmental changes, and bystander-associated effects warrant further investigation.

In addition to enhanced DNA damage and apoptosis, RT may also influence the tumor microenvironment and antitumor immune responses [21]. Previous studies have shown that RT can induce immunogenic cell death, alter cytokine signaling, and modulate immune cell infiltration within tumors [22, 23]. Emerging evidence also suggests that antibody–drug conjugates may exert immunomodulatory effects beyond direct cytotoxicity [24]. Therefore, the therapeutic efficacy observed following SG combined with RT may potentially involve additional microenvironmental or immune-related mechanisms that were not evaluated in the current study.

Although clinical validation is pending, the potent *in vivo* efficacy observed in our CDX and PDX models without concomitant weight loss supports the testing of SG–RT combinations in the neoadjuvant setting for CRC. Our observations are further supported by recent preclinical evidence in other epithelial malignancies. Zhou *et al.* [15] demonstrated that both SG and enfortumab vedotin synergize with ionizing radiation in bladder cancer models. A multicenter clinical study led by Alat *et al.* [25] reported that the combination of SG and RT in breast cancer significantly improved local control rates and objective responses, without increasing Grade ≥3 adverse events. These results provide a strong foundation for further clinical evaluation of SG combined with RT in locally advanced CRC, particularly in patients with high TROP2 expression. Given the manageable toxicity profile of SG, its incorporation into neoadjuvant RT regimens could potentially improve pathological response rates while maintaining treatment tolerability.

Despite these promising findings, several limitations of this study should be acknowledged. First, our study focused primarily on preclinical models; clinical validation is required to confirm the safety and therapeutic efficacy of SG combined with RT in patients with CRC. Second, while TROP2 expression appears to be critical for radiosensitization, its regulation in CRC microenvironments under radiation stress remains unclear. Third, because immunodeficient mouse models were used in the current study, the potential impact of treatment on the tumor immune microenvironment could not be evaluated. Notably, because the subcutaneous xenograft model used in this study did not expose the gastrointestinal tract to direct irradiation, the hematoxylin and eosin (H&E) findings (Supplementary Fig. S8) primarily reflect the absence of systemic drug-related mucosal toxicity and radiation-induced enteropathy as encountered in clinical pelvic irradiation settings cannot be fully assessed in this model. Future studies using immunocompetent orthotopic models will be important to further investigate the potential immunomodulatory effects and more comprehensively evaluate treatment-associated enteropathy and translational safety. Future investigations integrating single-cell transcriptomics and radiogenomic profiling may elucidate how TROP2 signaling interfaces with DNA repair and immune modulation pathways. Furthermore, the combination of SG with immune checkpoint inhibitors or DNA damage response inhibitors could provide synergistic therapeutic potential worthy of exploration.

This study revealed that the combination of SG and RT exhibited synergistic antitumor effects both *in vitro* and *in vivo.* Notably, the radiosensitizing effect of SG was dependent on TROP2 expression. These findings provide a compelling rationale for clinical trials evaluating SG-based combination strategies as a promising neoadjuvant therapeutic approach for locally advanced CRC.

## Supplementary Material

goag101_Supplementary_Data
