# Supplementary material for: Synergistic effects of sacituzumab govitecan and radiotherapy in preclinical models of colorectal cancer
Source: Gastroenterol Rep (Oxf). 2026 Oct 7;14:goag101. doi: 10.1093/gastro/goag101 (PMC13644529; doi:10.1093/gastro/goag101)
Supplement: Supplementary file 1 [file goag101_supplementary_data.zip › GR-2026-174.R1_Supplementary_figures+M.docx]

**Supplementary Fig. S1**

**Western blot detection of TROP2 levels of patient-derived xenograft (PDX) models.**

**Supplementary Fig. S2**

**SG synergizes with radiotherapy in DLD-1.**

**Supplementary Fig. S3**

**SG synergizes with radiotherapy in HCT116.**

**Supplementary Fig. S4**

**Representative macroscopic views of cell-line-derived xenograft (CDX) tumors from each treatment group.**

**NC, negative control; SG, sacituzumab govitecan; RT, radiotherapy.**

**Supplementary Fig. S5**

**Representative macroscopic views of patient-derived xenograft (PDX) tumors from each treatment group.**

**NC, negative control; SG, sacituzumab govitecan; RT, radiotherapy.**

**Supplementary Fig. S6**

**Western blot detection of TROP2 levels after the indicated treatments.**

**SG, sacituzumab govitecan; RT, radiotherapy.**

**Supplementary Fig. S7**

**Western blot detection of ABCG2 levels after radiotherapy (RT).**

**ABCG2, ATP-binding cassette super-family G member 2; RT, radiotherapy.**

**Supplementary Fig. S8**

**H&E staining in colonic tissues from CDX and PDX models treated with PBS control, SG, radiotherapy, or combination therapy (scale bar: 50 μm).**

**H&E, hematoxylin and eosin; CDX, cell-line-derived xenograft; PDX, patient-derived xenograft; NC, negative control; SG, sacituzumab govitecan; RT, radiotherapy.**
