# Supplementary material for: Synergistic effects of sacituzumab govitecan and radiotherapy in preclinical models of colorectal cancer
Source: Gastroenterol Rep (Oxf). 2026 Oct 7;14:goag101. doi: 10.1093/gastro/goag101 (PMC13644529; doi:10.1093/gastro/goag101)
Supplement: Supplementary file 1 [file goag101_supplementary_data.zip › GR-2026-174.R1_Supplementary_tables+M.docx]

**Supplementary Table S1**. Effect sizes and corresponding 95% confidence intervals for tumor growth inhibition in CDX models.

| Time point | Comparison | Mean difference | 95% CI | Adjusted *P* |
| --- | --- | --- | --- | --- |
| Day 21 | RT vs RT+SG | 216.8 mm³ | 47.5–386.1 | 0.0082 |
| Day 21 | SG vs RT+SG | 209.8 mm³ | 40.5–379.1 | 0.0110 |
| Day 24 | RT vs RT+SG | 265.4 mm³ | 96.1–434.7 | 0.0009 |
| Day 24 | SG vs RT+SG | 387.2 mm³ | 217.9–556.5 | < 0.0001 |
| Day 28 | RT vs RT+SG | 313.6 mm³ | 144.2–482.9 | < 0.0001 |
| Day 28 | SG vs RT+SG | 389.1 mm³ | 219.8–558.4 | < 0.0001 |
| Day 31 | RT vs RT+SG | 475.5 mm³ | 306.2–644.8 | < 0.0001 |
| Day 31 | SG vs RT+SG | 649.1 mm³ | 479.8–818.4 | < 0.0001 |

**Abbreviation**: CDX, cell-line-derived xenograft; SG, sacituzumab govitecan; RT, radiotherapy.

**Supplementary Table S2**. Effect sizes and corresponding 95% confidence intervals for tumor growth inhibition in PDX models.

| Time point | Comparison | Mean difference | 95% CI | Adjusted *P* |
| --- | --- | --- | --- | --- |
| Day 21 | RT vs RT+SG | 392.4 mm³ | 30.17–754.7 | 0.0282 |
| Day 21 | SG vs RT+SG | 284.7 mm³ | -77.51–647 | 0.1767 |
| Day 24 | RT vs RT+SG | 528.6 mm³ | 166.3–890.8 | 0.0013 |
| Day 24 | SG vs RT+SG | 193.8 mm³ | -168.5–556.1 | 0.5063 |
| Day 28 | RT vs RT+SG | 898.1 mm³ | 535.9–1260 | <0.0001 |
| Day 28 | SG vs RT+SG | 444 mm³ | 81.78–806.3 | 0.0095 |

**Abbreviation**: PDX, patient-derived xenograft; SG, sacituzumab govitecan; RT, radiotherapy.
